# Evaluation of the Impact of *Cordyceps cicadae* Mycelium on Vision Health: A Cohort Study

**DOI:** 10.7150/ijms.127003

**Published:** 2026-01-30

**Authors:** Lee Sun, Jui-Hsia Hsu, Yuan-Yen Chang, Chieh-Lin Wu, Min-Yen Hsu, Yu-Chien Hung, Chin-Chu Chen, Hui-Wen Lin

**Affiliations:** 1 Department of Optometry, Asia University, Taichung 413, Taiwan.; 2 Biotech Research Institute, Grape King Bio Ltd, Taoyuan City, Taiwan.; 3 Department of Microbiology and Immunology, School of Medicine, Chung Shan Medical University and Clinical Laboratory, Chung Shan Medical University Hospital, Taichung, Taiwan.; 4 Save Sight Institute, The Faculty of Medicine and Health, The University of Sydney, Sydney, NSW 2000, Australia.; 5 Department of Optometry, Chung Shan Medical University, Taichung 40201, Taiwan.; 6 Department of Ophthalmology, Chung Shan Medical University Hospital, Taichung, Taiwan.; 7 School of Medicine, Chung Shan Medical University, Taichung 40201, Taiwan.; 8 Biotechnology Center, National Chung Hsing University, Taichung 40201, Taiwan.; 9 Institute of Food Science and Technology, National Taiwan University, Taipei City, Taiwan.; 10 Institute of Biopharmaceutical Science, National Sun Yat-sen University, Kaohsiung, Taiwan.; 11 Department of Bioscience Technology, Chung Yuan Christian University, Taoyuan City, Taiwan.; 12 Institute of Medicine, Chung Shan Medical University, Taichung, Taiwan.; 13 Department of Medical Research, Chung Shan Medical University Hospital, Taichung, Taiwan.

**Keywords:** *Cordyceps cicadae* mycelium (CCM), visual acuity, ocular fatigue, eye strain

## Abstract

*Cordyceps cicadae* is a traditional Chinese medicinal fungus known for its diverse bioactive compounds and pharmacological properties similar to those of *Cordyceps sinensis*. Extracts of *C. cicadae* have been reported to alleviate dry eye syndrome, reduce intraocular pressure (IOP), and show therapeutic potential in various ocular diseases. In this study, we evaluated the effects of *C. cicadae* mycelium (CCM) intake on visual function in 60 healthy volunteers. Comprehensive eye examinations including visual acuity, axial length, corneal curvature, and refractive error were conducted at six time points: baseline (day 0, pre-intake), one- and two-hours post-intake on day 0 and again on day 28 (pre-final intake and one- and two- hours post-intake), following a 28-day period of continuous supplementation. Subjective ocular surface condition was also assessed using an OSDI questionnaire. No significant changes were observed in axial length, corneal curvature, or refractive error throughout the study. However, improvements in VA were noted in a subset of participants as early as one hour after CCM intake, with further enhancement at two hours and sustained improvement following 28 days of daily supplementation. Subjective reports also indicated a marked reduction in eye fatigue after CCM consumption. These findings suggest that CCM supplementation may serve as a supportive strategy for relieving digital eye strain and enhancing visual function.

## Introduction

*Cordyceps cicadae* (*C. cicadae*) is an entomopathogenic fungus that parasitizes the larvae of various Cicada species 1. The mycelium consumes the host body and produces blossom-like stromata, primarily found in humid mountainous regions of Asia at low altitudes ranging from 80 to 500 meters. *C. cicadae* have long been regarded as a traditional Chinese herbal medicine, utilized in a wide range of therapeutic applications. Its reported pharmacological properties included anti-bacterial 2, anti-cancer 3, 4, anti-inflammatory 5, anti-oxidative and anti-aging effects 6-8. It has also been associated with the regulation of blood cholesterol and glucose levels 9-11, as well as renal protection 12, 13 and hepatic protection 14, among other biological functions.

Previous studies have demonstrated that *C. cicadae* contain various bioactive compounds 15, including adenosine, ergosterol, and polysaccharides. Among these, N6-(2-hydroxyethyl) adenosine (HEA) has been identified as a major active component purified from* C. cicadae* 16. HEA has been shown to confer protective effects against H_2_O_2_-induced oxidative damage in PC12 cells 17. In addition, HEA exhibits anti-inflammatory properties 18, reduces reactive oxygen species (ROS) levels, and displays potent antioxidant activity 19. It also possesses immunomodulatory and neuroprotective functions 20. Notably, a previous study confirmed that HEA could significantly reduce intraocular pressure (IOP) within 90 minutes of administration 21. Furthermore, HEA has been shown to cross the blood-brain barrier (BBB) 22; suggesting the possibility that it may also penetrate the blood-retina barrier (BRB) and exert effects on ocular tissues within a short timeframe.

In this study, *C. cicadae* mycelium was obtained through liquid-state fermentation and subsequently processed into a lyophilized powder 23, provided by Grape King Bio Ltd. Precursor studies have demonstrated that HEA exhibited no cytotoxicity in cultured CHO-K1 cells 24 and does not induce hepatic toxicity in animal models, including rats and rabbits 25-27. Moreover, oral administration of HEA-enriched *C. cicadae* mycelium is safe for the central nervous, cardiovascular, and respiratory systems in mice 22. A clinical trial involving 49 healthy volunteers evaluated the safety of daily oral intake of *C. cicadae* fermentation-derived mycelium powder capsules over a three-month period. Blood biochemical analyses revealed no evidence of hepatic or renal toxicity 28. In a separate clinical study, the same formulation was administered for three months to 22 volunteers with hyperglycemia or type 2 diabetes. The results demonstrated its efficacy in blood glucose regulation, with no reported adverse effects or signs of liver or kidney dysfunction 29.

The therapeutic application of *C. cicadae* in ocular diseases has been documented in several ancient Chinese medical texts, where it was traditionally employed in the treatment of conditions such as acute conjunctivitis, chronic blepharitis, chronic dacryocystitis, and pterygium. In modern studies, *C. cicadae* have been extensively investigated for its potential benefits in various eye disorders. Notably, it has demonstrated the ability to reduce intraocular pressure in both glaucoma patients and animal models 21, 27, 30, prevent UVB-induced cataract formation 31, and alleviate symptoms of dry eye disease 32. Additionally, *C. cicadae* have been reported to possess anti-fatigue properties 33. In this study, we aimed to evaluate whether oral supplementation with *C. cicadae* mycelium (CCM) powder could alleviate symptoms of asthenopia (visual fatigue) and improve visual function over a 28-day observational period. Post-intervention assessments included comprehensive eye examinations, such as visual acuity testing, measurement of refractive error using an autorefractor, corneal curvature assessment via keratometry, and axial length determination using the noninvasive optical biometer, Lenstar. Moreover, a structured questionnaire was administered to collect subjective feedback from participants regarding their ocular comfort and visual performance following daily intake of CCM capsules.

## Materials and Methods

### Sample Preparation and High-Performance Liquid Chromatography (HPLC) Analysis

The *C. cicadae* MU 30,106 strain, obtained from the Bioresource Collection and Research Center (BCRC; Hsinchu, Taiwan), was initially cultured on potato dextrose agar at 25 °C for 7 days. Subsequently, the culture was transferred into a 500-mL Hinton flask containing 250 mL of broth and incubated under shaking conditions (120 rpm, 25 °C) for 3 days. The resulting broth containing *C. cicadae* cells was then inoculated into a 500-L fermenter (working volume: 350 L; agitation: 60 rpm; aeration: 0.3 vvm; temperature: 25 °C) for 3 days, followed by scale-up to a 20-ton fermenter (containing 16 tons of broth; agitation: 30 rpm; aeration: 0.3 vvm; temperature: 25 °C) for an additional 5 days. The fermentation broth consisted of 1% soybean powder, 5% glucose, and 1% yeast extract, adjusted to pH 6.0. After fermentation, the mycelium was harvested and subsequently lyophilized into a powder form. Each capsule contained 250 mg of mycelium powder mixed with 20 mg of excipients (magnesium stearate, Avicel PH-301, and silicon dioxide), resulting in 270 mg per capsule. Two capsules constituted a single serving. The capsule shells were composed of gelatin, purified water, sodium lauryl sulfate, and glycerin. Chemical analysis of the mycelium powder was conducted using high-performance liquid chromatography (HPLC). The bioactive compounds detected were adenosine and N6-(2-hydroxyethyl) adenosine (HEA), with a retention time of 14.9 minutes (Supplementary Figure 1). The HEA content in the *C. cicadae* mycelium powder was determined to be 2.5 mg/g.

### Study Design and Procedures

The trial was approved by the Institutional Review Board of China Medical University & Hospital Research Ethics Center, Taichung, Taiwan (IRB No. CRREC-111-087), and was registered at ClinicalTrials.gov (Date: 08/03/2023; Identifier: NCT05778409; https://register.clinicaltrials.gov). This study was conducted following the Declaration of Helsinki. Volunteers were recruited from the student population of Asia University (Taichung, Taiwan). A total of 60 participants, consisting of 20 males and 40 females, were enrolled. All were Taiwanese students aged between 20 and 25 years. Individuals with active eye infection, myopia greater than -7.00 diopters (spherical equivalent), or an inability to comply with the examination procedures were excluded at the time of enrollment period. Prior to participation, all subjects received a full explanation of the study and provided written informed consent, obtained by the principal investigator.

The study used a within-subject design, with each participant serving as their own control. Prior to the intervention, participants were instructed to discontinue any previously used dietary supplements. All eye examinations were conducted at the Optometry Laboratory of Asia University. Measurements were taken at baseline (prior to CCM intake) on day 0, and at one- and two-hours post-intake. The study included a 28-day follow-up period, during which participants were instructed to take two CCM capsules daily. They were required to return for follow-up assessments on day 28 after the initial intake, and collect eye examination data before and one and two hours after the last CCM intake. All data were documented in case report forms.

### Eye Assessment

Prior to CCM administration on day 0, all participants underwent baseline ocular examinations, including autorefraction, axial length measurement, corneal curvature assessment, and distance visual acuity (VA) testing. Refractive status, including spherical and cylindrical powers, was evaluated using an autorefractor (NVision-K 5001, Shin-Nippon, Japan) without pharmacological cycloplegia. Axial length was measured with a noninvasive optical biometer (LENSTAR 900 optical biometer, Haag-Streit, Switzerland). Corneal curvature assessed via keratometry (OM-4, Topcon, Japan), with measurements obtained from the two principal meridians, vertical and horizontal. Corneal astigmatism was calculated as the difference between these two meridians. All ophthalmic instruments were calibrated according to the manufacturers' recommendations prior to the study and underwent routine maintenance and performance checks throughout the study period.

Distance VA was assessed at a distance of 6 meters using a standard Snellen chart and recorded in LogMAR notation for the right eye (OD), left eye (OS), and both eyes (OU). All visual acuity tests in this study were conducted using the same standardized Snellen chart under fixed illumination (approximately 300-350 lux) and at a fixed testing distance. The lighting source, room setup, and seating height were kept consistent throughout the measurements.

After the preliminary assessment of ocular status, participants were instructed to take the CCM. The same set of examinations as conducted at baseline was then repeated one and two hours after intake on the same day. Follow-up assessments were conducted 28 ± 3 days later. Participants returned to the laboratory, and the aforementioned ocular assessments were repeated prior to the final CCM intake, as well as at one and two hours after the last dose.

All ocular examinations were performed by senior undergraduate students from the Department of Optometry at Asia University who had passed the clinical skills proficiency test. To ensure consistency throughout the study, each participant was examined by the same examiner at all time points.

### Ocular Surface Condition Questionnaires Survey

In this investigation, the Ocular Surface Disease Index (OSDI) questionnaire was administered to evaluate participants' ocular condition and subjective symptoms following CCM intake. Responses were collected at baseline, as well as one- and two-hours post-intake on day 0, and again during the 28-day follow-up period, providing a comprehensive assessment of changes in ocular comfort and visual symptoms over time. In this section, the OSDI questionnaire was also analyzed to evaluate improvements in ocular surface comfort following CCM intake. Scores were converted into percentages to facilitate comparison.

### Statistical Analysis

Data were analyzed using GraphPad Prism (GraphPad Software, San Diego, CA, USA) and SPSS (IBM SPSS Statistics, Armonk, NY, USA). All results are presented as mean ± Standard Error of the Mean (SEM). Statistical analyses included paired Student's *t*-tests and repeated measures analysis of variance (ANOVA) with Bonferroni multiple comparison tests to evaluate changes before and after CCM intake. Day and time effects were further assessed using a mixed-effects model for repeated measures. A p-value of < 0.05 was considered statistically significant. All participants were included in the final statistical analysis (n = 60).

### Safety Assessment

During the trial, the principal investigator and co-investigators will be present on site to actively monitor and assess participant safety after intake. This study involves only non-invasive ophthalmic examinations using professional diagnostic instruments, and no invasive medical procedures are performed. Therefore, the potential risk of adverse effects and their incidence are considered to be very low. In the event that any adverse or allergic reactions occur after intake, the participant will be immediately referred to Asia University Hospital for appropriate management and necessary medical care.

## Results

### Study Subjects

**Figure 1:**
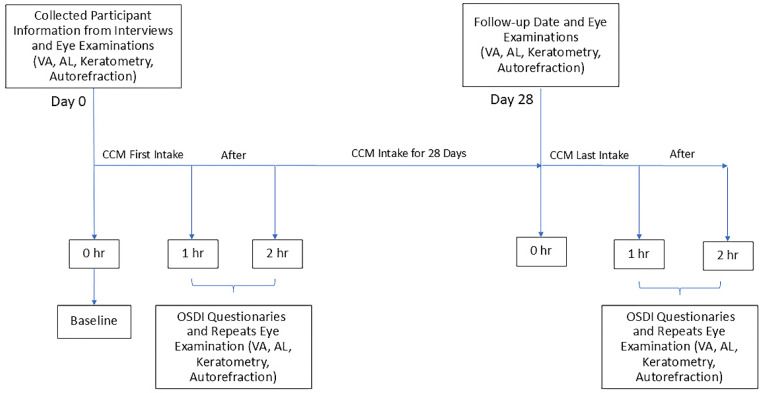
** Timeline of CCM treatment and corresponding eye examination time points.** Eye examinations were conducted at baseline (the first visit) and repeated at 1 and 2 hours after the initial CCM intake. After 28 days of continuous CCM supplementation, participants returned for a follow-up examination. On the final day of intake, eye examinations were performed again at pre-intake, as well as one- and two- hours post-intake. At each time point, participants underwent assessments including visual acuity (VA), axial length (AL), keratometry, and autorefraction. Additionally, the Ocular Surface Disease Index (OSDI) questionnaires were administered to evaluate subjective ocular fatigue following CCM intake.

A total of 60 college students aged 20 years or older participated in the study, including 40 females and 20 males. All participants received oral CCM supplementation daily for 28 consecutive days. To evaluate the effects of the intervention, ocular examinations were performed at six time points: on day 0, prior to CCM intake as baseline, and at one- and two-hours post-intake; and also on day 28, again before the final intake and at one and two hours afterward. The same set of assessment parameters was used across all time points. The experimental procedure is illustrated in Figure 1. During the study, adverse events were proactively assessed and recorded by the research staff, and no study-related adverse reactions were observed.

### Effects of Cordyceps cicadae Mycelium on Axial Length, Corneal Curvature, and Refractive Status

To evaluate whether the consumption of CCM induced alterations in ocular physiology and refractive status, a series of noninvasive ophthalmic assessments were conducted. Ocular parameters, including axial length, corneal curvature, and refractive error, were measured at six time points before the first and after the final intake of CCM-throughout the 28-days study period. All data were analyzed using repeated measures ANOVA to assess the effect of time, and a mixed-effects model was applied to further evaluate longitudinal trends.

**Figure 2:**
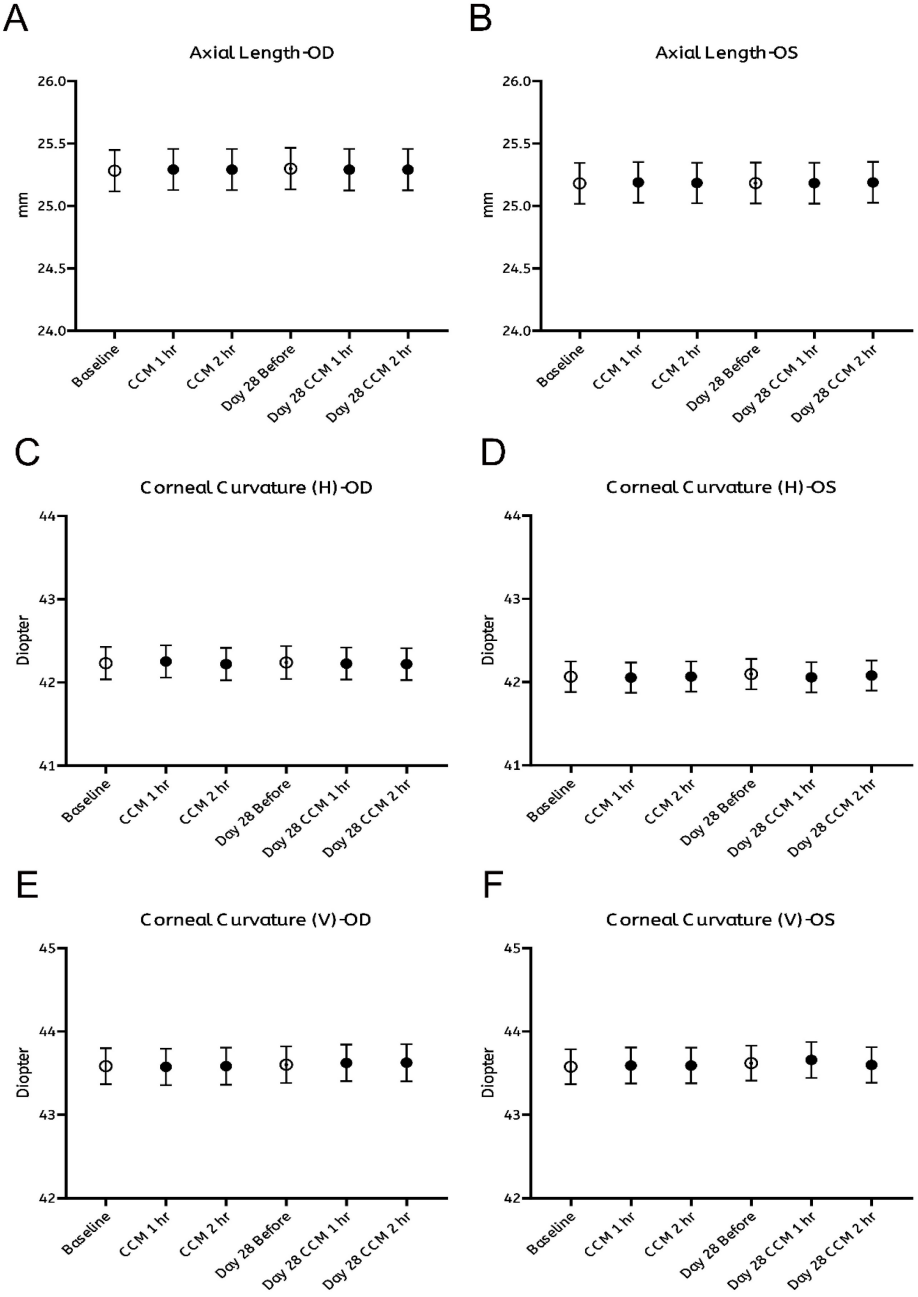
** Assessment of axial length and corneal curvature before and after CCM intake. (A, B)** Axial length measured using a non-invasive optical biometer (Lenstar) at baseline (0 hours, prior to CCM intake), 1 and 2 hours post-intake, with the same measurements repeated on day 28 at corresponding time points for right eye (OD, A) and left eye (OS, B). **(C-F)** Keratometry measurements of corneal curvature at the same timepoints, showing the horizontal meridian for right eye (C) and left eye (D), and the vertical meridian for right eye (E) and left eye (F). All results are presented as mean ± standard error of the mean (SEM). Statistical analyses were performed using paired Student's *t*-test and repeated measures analysis of variance (ANOVA) with Bonferroni correction for multiple comparisons to evaluate changes before and after CCM intake. No significant differences in axial length, corneal curvature were observed throughout the 28-day follow-up period.

**Figure 3:**
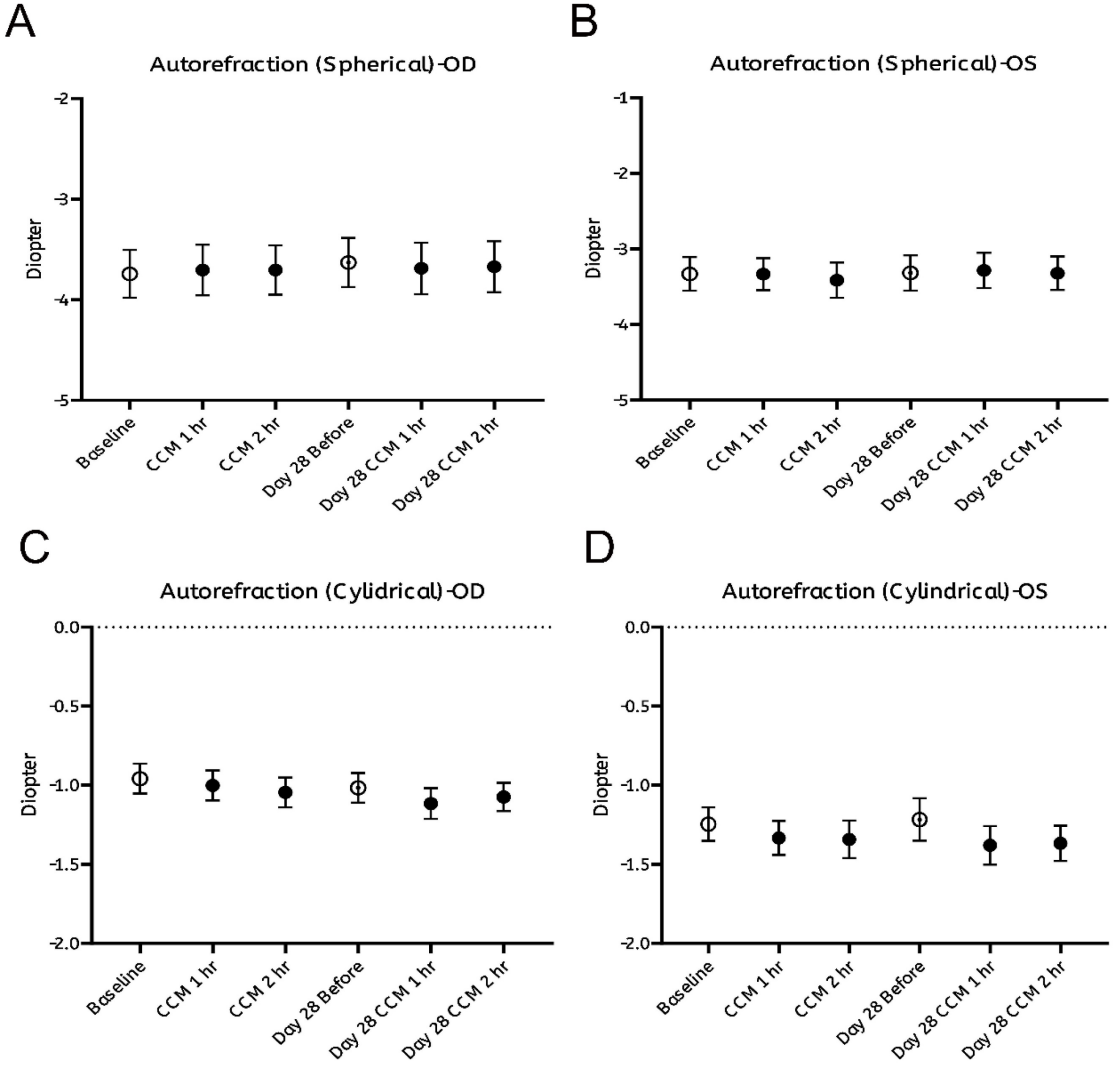
** Assessment of refractive status before and after CCM intake. (A, B)**. Spherical and **(C, D)** cylindrical diopter power measured using an autorefractor without pharmacological cycloplegia at the same time points for right eye (A, C) and left eye (B, D). All results are presented as mean ± standard error of the mean (SEM). Statistical analyses were performed using paired Student's *t*-test and repeated measures analysis of variance (ANOVA) with Bonferroni correction for multiple comparisons to evaluate changes before and after CCM intake. No significant differences in refractive status were observed throughout the 28-day follow-up period.

As summarized in Figure 2, no significant changes were observed in any of the measured ocular biometric parameters, including axial length (Figure 2A, B) and horizontal or vertical corneal curvature (K-readings; Figure 2C-F). Similarly, refractive error, expressed in spherical (Figure 3A, B) and cylindrical (Figure 3C, D) degrees, remained stable throughout both the intervention and follow-up phases. These findings indicate that CCM consumption did not produce measurable changes in ocular biometry or refractive status during the 28-day observation period.

### Improvements in Visual Acuity Following Cordyceps cicadae Mycelium Intake

**Figure 4:**
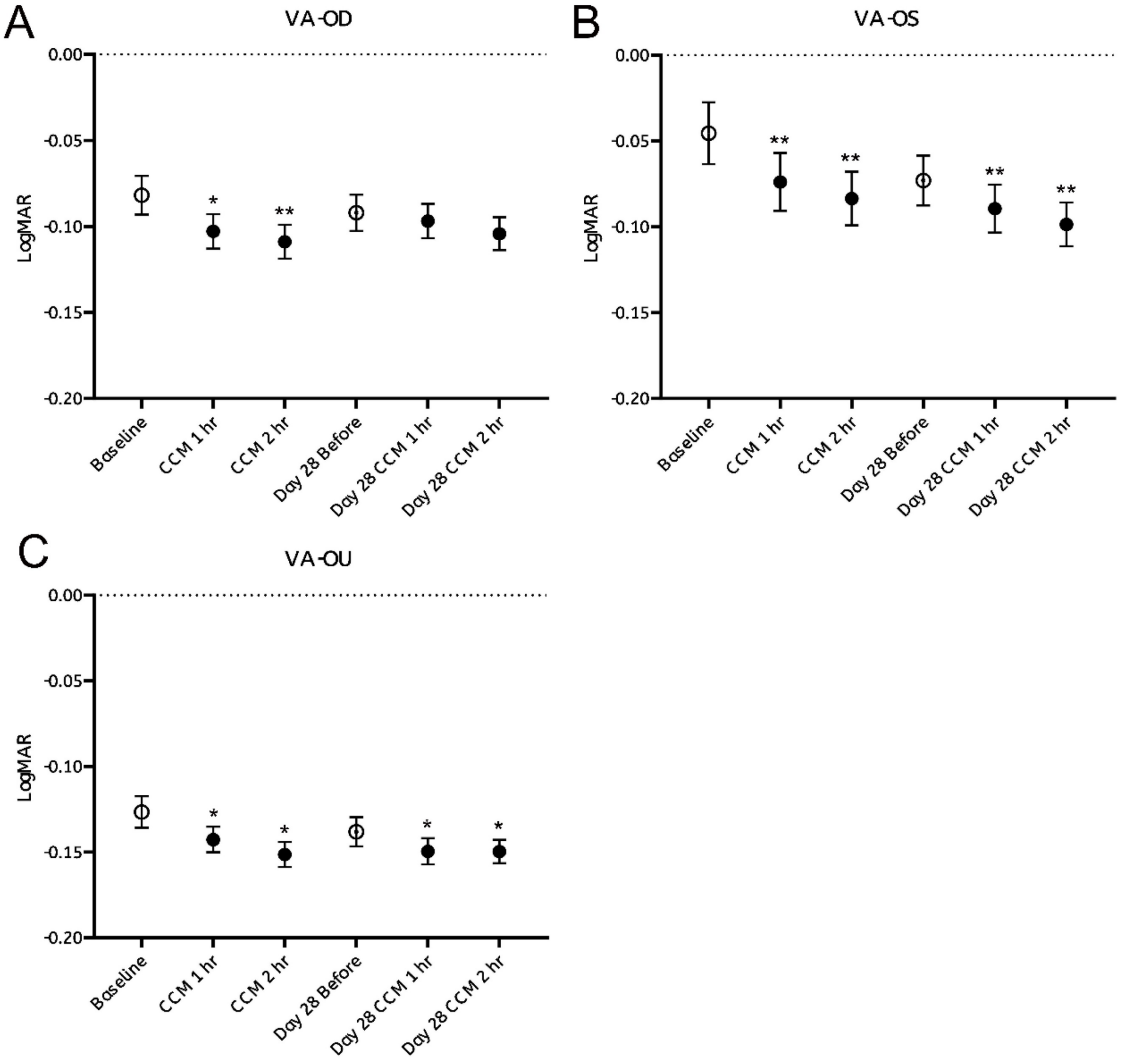
** Visual acuity assessment before and after CCM consumption. (A-C)** Visual acuity test results were converted to LogMAR notation, and changes at each time point before and after CCM intake were evaluated over a 28-day follow-up period. (A) Right eye (OD); (B) Left eye (OS); (C) Binocular (OU). Statistical analysis was performed using repeated measures analysis of variance (ANOVA). Statistical significance was denoted as *p < 0.05 and **p < 0.01; the absence of asterisks denotes non-significant differences.

Visual acuity (VA) was assessed by comparing pre- and post-intake performance on the Snellen chart, with results expressed in LogMAR units. Approximately 40% of participants exhibited measurable improvements within the first hour following CCM administration in VA, recognizing on average more than one additional optotype per line, corresponding to a significant reduction in LogMAR values (Figure 4A-C, black dots). Notably, participants who demonstrated bilateral VA enhancement also showed concurrent improvement in at least one eye (OD or OS), suggesting that monocular gains contributed to the observed binocular improvement.

These improvements were sustained at the second hour post-intake, with a new subset of participants—who had shown no measurable change during the first hour—displaying delayed-onset VA enhancement. These findings indicate that some participants exhibited measurable improvements in both monocular and binocular VA following CCM intake on the first day.

At the follow-up examination after 28 days of continuous CCM supplementation, VA values assessed prior to the final dose on day 28 remained elevated relative to the initial baseline (Figure 4A-C, white dots). Additional improvements in VA were observed one- and two- hours after the final administration, as reflected by further reduction in LogMAR values. These observations indicate that some participants continued to exhibit improvements in visual acuity throughout the 28-day intervention period.

However, the magnitude of improvement appeared to reach plateau, as VA value measured after CCM intake on day 28 nearly identical to those observed following the initial administration on day 0 (Figure 4A-C, black dots). Statistically significant changes were observed in the left eye and binocular VA, while the right eye showed a similar trend that did not reach statistical significance. Repeated measures ANOVA confirmed a significant main effect of time on visual acuity (VA) following the initial CCM administration (Supplementary Table 1).

Overall, these findings indicate that CCM supplementation yields measurable improvements in VA in a substantial proportion of participants, results in both transient and sustained benefits, with continued but limited gains rather than progressively increasing effects.

### Impact of Refractive Error on Visual Acuity Improvement Following Cordyceps cicadae Mycelium Intake

To evaluate whether participants' refractive status correlates with the amount of VA improvement following CCM intake, all participants were categorized into three groups based on myopic severity: low myopia (< -3.00 D), moderate myopia (-3.00 D to -5.00 D), and high myopia (> -5.00 D). A fixed-effects model was employed to assess the association between refractive error and VA improvement.

**Figure 5:**
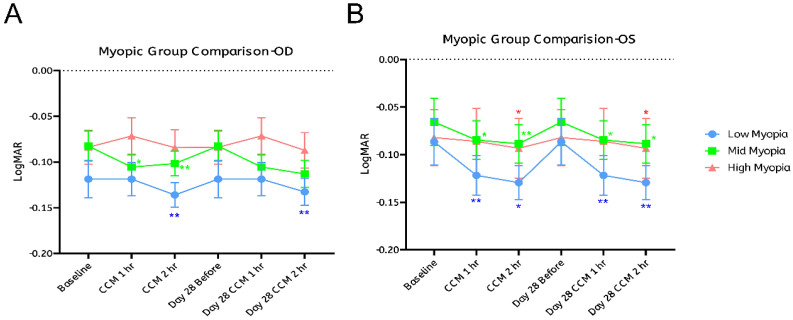
** Visual acuity improvement stratified by degree of myopia.** Participants were categorized into three groups based on refractive error: low myopia (< -3.00 D, shown in blue), moderate myopia (-3.00 D to -5.00 D, shown in green), and high myopia (> -5.00 D, shown in red). The potential influence of myopia severity on visual acuity (VA) improvement was evaluated separately for the right eye (OD, A) and the left eye (OS, B). A fixed-effects model was applied to examine the association between refractive error and VA improvement. Sample sizes for each subgroup were as follows: low myopia—OD: n = 15, OS: n = 17; moderate myopia—OD: n = 24, OS: n = 28; high myopia—OD: n = 21, OS: n = 15. Statistical significance was indicated as *p < 0.05 and **p < 0.01; the absence of asterisks denotes non-significant differences.

We next analyzed each refractive subgroup separately. In the low myopia group, the left eye showed statistically significant improvements at both one- and two- hours post-intake on day 0, which were maintained through day 28 (Figure 5B, blue dots). The right eye showed significant improvement only at the second hour after CCM intake on both day 0 and day 28 (Figure 5A, blue dots). Although first-hour changes on both days were not statistically significant.

In the moderate myopia group, the left eye consistently showed significant improvements at both one- and two-hours post-intake on days 0 and 28 (Figure 5B, green dots). The right eye showed significant gains only on day 0 at both one- and two-hours post-intake; on day 28, changes were not statistically significant, although LogMAR values exhibited a downward trend (Figure 5A, green dots). In the high myopia group, no significant changes were observed in the right eye at any time point (Figure 5A, red dots), while the left eye showed improvement only at the second hour post-intake on both days 0 and 28 (Figure 5B, red dots).

These findings indicate that the temporal patterns of VA changes following CCM intake were generally similar across eyes and refractive subgroups, but the extent of changes varied. The left eye showed consistent, time-dependent improvements, with statistically significant gains observed on both day 0 and day 28 in all three subgroups. In contrast, the right eye exhibited more limited changes: significant improvements were observed only in the low myopia group at the two-hour mark on both days, while no significant changes were detected in the high myopia group, and the moderate myopia group showed no significant improvement on day 28.

Although the observed patterns indicate somewhat greater changes in participants with lower degrees of myopia, statistical analysis revealed no significant differences between the refractive subgroups. These results suggest that the degree of myopia may not be a strong predictor of response, despite trends showing relatively larger changes in the low and moderate myopia groups.

### Ocular Surface Condition Questionnaires

**Figure 6:**
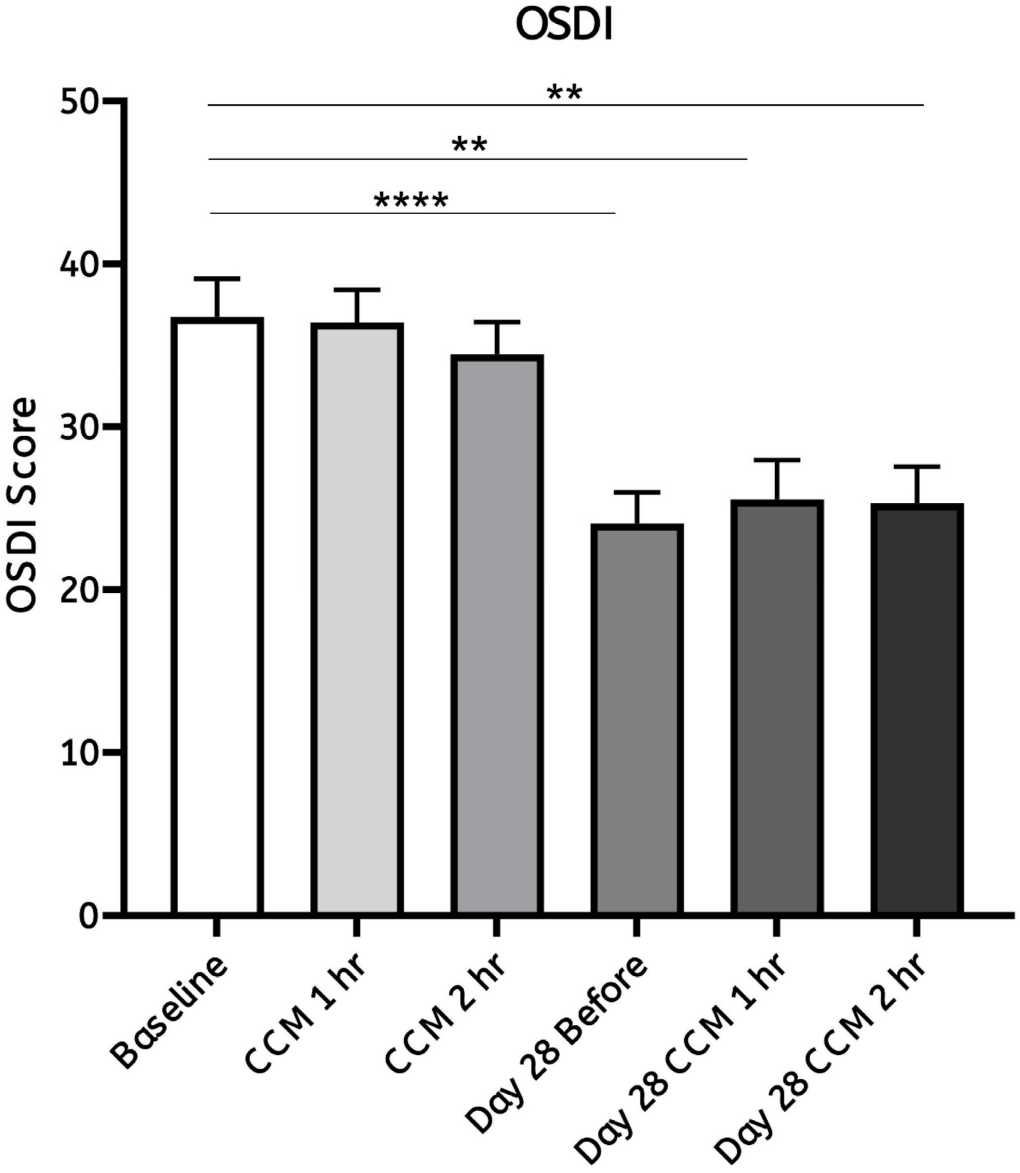
** Statistical analysis of ocular fatigue assessed by the OSDI questionnaire following CCM intake.** Results from the Ocular Surface Disease Index (OSDI) questionnaires were used to assess participants' ocular condition and subjective symptoms before the initial (day 0) and after the final (day 28) CCM intake. On day 0, no significant changes were observed at either time point. By day 28, notable improvements were observed, with the average OSDI score decreasing, reflecting symptom reductions at the corresponding time points during the 28-day follow-up period. Statistical analysis was performed using a paired Student's *T*-test. Significance is indicated as **p < 0.01 and ****p < 0.0001; the absence of asterisks denotes non-significant differences.

Participants' subjective experiences were assessed using the Ocular Surface Disease Index (OSDI) 34 questionnaires, a validated tool for evaluating ocular discomfort and dry eye symptoms, following CCM intake. The average OSDI score decreased from 36.73 on day 0 (baseline) to 24.05 on day 28 (Figure 6). These results indicate a notable reduction in visual discomfort during daily activities, indicating a reduction in self-reported ocular discomfort and dry eye-related symptoms during daily activities. Participants were asked to complete the OSDI questionnaire to assess ocular fatigue at each time point following CCM intake on day 0 and day 28. On day 0, no significant changes in OSDI scores were observed at either one- or two- hours after CCM intake. In contrast, by day 28, OSDI scores showed a marked decrease, with both the one- and two-hour post-intake scores being lower than those recorded on day 0. These findings suggest a progressive improvement in ocular fatigue over the 28-day follow-up period.

In addition, subjective assessments of visual quality at the end of the 28-day period showed that 53.3% of participants reported slight improvement, 10% reported marked improvement, and 36.7% reported no noticeable change in their visual experience. Participants' lifestyles were monitored throughout the study using self-administered questionnaires. No significant changes were observed in sleep duration (mean: 6.7 ± 1.0 hours/day), mobile phone usage (mean: 5.9 ± 1.0 hours/day), or computer usage time (mean: 3.18 ± 0.25 hours/day). Adherence to the daily CCM intake regimen was high, with nearly 80% of participants reporting consistent compliance, while approximately 18% reported occasional lapses due to forgetfulness.

## Discussion

In this study, a 28-day follow-up trial was conducted to evaluate the effects of CCM intake on visual acuity, axial length, corneal curvature, and refractive status at specific time points before and after consumption on day 0 and day 28. No significant changes were observed in ocular biometric parameters, including axial length, corneal curvature, or refractive status throughout the study period. Notably, more than one-third of participants showed measurable improvements in VA within the first hour after CCM intake, with the number of responders increasing by the second hour. Some participants also demonstrated VA enhancement on day 28. These findings suggest that improvements in VA observed after CCM intake may be maintained over the 28-day intervention period.

Among all participants, three individuals exhibited marked bilateral VA improvement within the first hour following CCM intake, with gains sustained throughout the second hour and maintained throughout the 28-day intervention period. In contrast, four participants showed no early response but demonstrated significant bilateral VA improvements only after 28 days of continuous supplementation, with benefits persisting through the end of the study. Among the remaining participants, unilateral improvements were more common, although a substantial number still showed measurable VA gains by day 28. Notably, nine participants exhibited no detectable change in VA at any time point during the study.

In summary, CCM intake was associated with both immediate and long-term improvements in visual acuity, with response patterns varying among individuals. Bilateral enhancement was relatively uncommon, as most participants exhibited monocular gains. It remains uncertain whether the eye showing greater improvement corresponded to ocular dominance. These results suggest a potentially asymmetric or eye-specific response to CCM, emphasizing the need for further investigation into the roles of ocular dominance and interocular variability in future studies.

Although incorporating a placebo or vehicle control group would have allowed a more objective comparison, however, variability among individuals may also be present. This study employed a within-subject design to intuitively monitor changes following CCM intake and to minimize inter-individual differences. Moreover, as no invasive procedures or cycloplegia were involved, no blood sampling was performed. As for hepatic and renal toxicity, previous studies have provided sufficient evidence supporting the safety of CCM 28-30, 35. All assessments relied on subjective visual acuity tests and questionnaire evaluations, which are likely to provide a more direct reflection of participants' immediate visual responses to CCM intake.

In a study by Hsu *et al*. 30, CCM was shown to rapidly reduce elevated intraocular pressure (IOP) in most individuals within 90 minutes of administration. These findings suggest that the active components of *C. cicadae* can be quickly delivered to ocular tissues, which is consistent with our observations of visual acuity progression at one- and two-hours following CCM intake. In the present study, we posit that the anti-fatigue effects and IOP-lowering properties of CCM may collectively contribute to improvements in participants' vision. Although reductions in IOP are not necessarily associated with changes in retinal artery blood flow 36, they can facilitate enhanced aqueous humor drainage, thereby influencing ocular homeostasis. Furthermore, previous animal studies have highlighted oxidative stress as a critical factor in the development of elevated IOP and glaucoma-related lesions. Antioxidant compounds are thus considered to play a significant role in mitigating IOP elevation and associated ocular pathologies 27. Based on these findings, the antioxidant properties of CCM may also contribute to its observed ocular benefits.

Several studies have suggested that agonists of adenosine receptors, such as A1, A2A, and A3, can induce an increase in intracellular Ca²⁺ levels, leading to a reduction in the size of trabecular meshwork cells. This morphological change facilitates the outflow of aqueous humor and subsequently reduces IOP 37. HEA found in *C. cicadae* is an adenosine analog, and previous animal studies have confirmed its ability to lower IOP in rabbit models 30.

Additionally, one potential contributor to aqueous humor outflow is the contractile activity of the ciliary muscle. Tendinous fibers extending from the anterior region of the ciliary muscle pass through the trabecular meshwork and insert into the walls of Schlemm's canal. Contraction of the ciliary muscle can increase the number of pores within the trabecular meshwork, thereby facilitating aqueous humor drainage into Schlemm's canal and leading to a rapid reduction in IOP 38. Whether the observed improvements in VA in this study are associated with the modulation of ciliary muscle-trabecular meshwork interactions by active components in CCM remains unclear and warrants further investigation.

Although significant VA improvements following CCM intake were observed over time across all refractive error groups, VA responses varied across myopic subgroups. Participants with low and moderate myopia consistently demonstrated the most pronounced improvement, whereas those with high myopia showed minimal or delayed effects. However, a post hoc analysis was conducted using the McNemar chi-square test to further validate the effects. Overall, no significant differences were observed between refractive subgroups. Consistent with this, results from the mixed-effects model indicated that the differences among subgroups were not statistically significant, suggesting that the degree of myopia did not significantly correlate with the magnitude of VA improvement. In contrast, time had a more pronounced effect on both the right and left eyes, highlighting the temporal dynamics of visual acuity improvement following CCM administration. This finding may be partly attributed to the limited sample sizes in each subgroup (low myopia: OD n = 15, OS n = 17; moderate myopia: OD n = 24, OS, n = 28; high myopia: OD n = 21, OS n = 15), which may have reduced statistical power and limited the ability to detect significant differences.

Dry eye symptoms were evaluated using the Ocular Surface Disease Index (OSDI) 34, a validated questionnaire that assesses the frequency and severity of ocular discomfort, visual disturbance, and environmental triggers affecting daily life. Among our predominantly college student participants, prolonged use of computers and mobile devices, as well as extended near work, may have contributed to ocular discomfort and fluctuating vision, both of which are common manifestations of tear film instability.

The present findings suggest that CCM intake may help alleviate subjective dry eye symptoms and eye strain, which could in turn contribute to improved visual acuity. Previous studies have demonstrated the anti-fatigue 33. and ocular surface-protective properties of *Cordyceps cicadae* extracts 39, and a clinical trial has reported that CCM supplementation can relieve dry eye symptoms 32. Other studies have suggested that the minimal clinically important difference (MCID) for the OSDI ranges from 4.5 to 7.3 for mild or moderate disease and from 7.3 to 13.4 for severe disease 40. Based on our experimental results, the mean OSDI score decreased by 12.68 points, which exceeds the commonly reported MCID range, indicating a potentially clinically meaningful improvement in dry eye symptoms. Given that tear film stability directly influences optical quality 41, it is plausible that CCM enhances visual performance by improving tear film integrity. Further studies are warranted to elucidate the precise mechanisms underlying these effects.

In conclusion, the findings of this study suggest that CCM intake may help alleviate eye strain and improve visual acuity. Further research is warranted to elucidate the underlying mechanisms, including potential effects on ciliary muscle function, accommodative performance, and tear film stability. Future investigations are planned to assess these aspects through standard clinical tests—such as near point of accommodation (NPA), fused cross cylinder (FCC), and accommodative facility testing with ±2.00 D flipper lenses—as well as to evaluate changes in choroidal thickness via optical coherence tomography (OCT) and quality through tear breakup time (TBUT) measurements. Age-related differences will also be considered to determine whether improvements in ocular function vary across different age groups. Collectively, these studies aim to clarify the broader potential of CCM as a supportive intervention for maintaining ocular health.

## Supplementary Material

Supplementary figure and table.

## Data Availability

The datasets used and/or analyzed during the current study are available from the corresponding author on reasonable request.
