# Supplementary material for: Evaluation of the Impact of Cordyceps cicadae Mycelium on Vision Health: A Cohort Study
Source: Int J Med Sci. 2026 Jan 30;23(3):864–75. doi: 10.7150/ijms.127003 (PMC12964581; doi:10.7150/ijms.127003)
Supplement: Supplementary file 1 — Supplementary figure and table. [file ijmsv23p0864s1.pdf]

Supplementary Table 1

	Day 0		Day 28		
	Mean	SD	Mean	SD	*P*
LogMAR OD					0.002
0 hr	-0.096	0.093	-0.106	0.088	
1 hr	-0.120*	0.081	-0.110	0.082	
2 hr	-0.123**	0.080	-0.118	0.079	
logMAR OS					<0.001
0 hr	-0.058	0.145	-0.088	0.123	
1 hr	-0.087**	0.135	-0.109**	0.113	
2 hr	-0.096**	0.125	-0.115**	0.107	
logMAR OU					0.014
0 hr	-0.145	0.076	-0.156	0.069	
1 hr	-0.161*	0.059	-0.165*	0.063	
2 hr	-0.164*	0.068	-0.166*	0.056	

Table 1. Changes in monocular and binocular visual acuity before and at 1 and 2 hours after CCM intake on day 0 and day 28. Visual acuity was measured in LogMAR units at baseline (0 hr), 1 hour, and 2 hours post-CCM intake on both day 0 and day 28. P-values represent the time effect analyzed by repeated measures ANOVA. *p < 0.05; **p < 0.01 indicate significant differences from baseline (0 hr on day 0) using Bonferroni correction for multiple comparisons.

Supplementary Figure 1

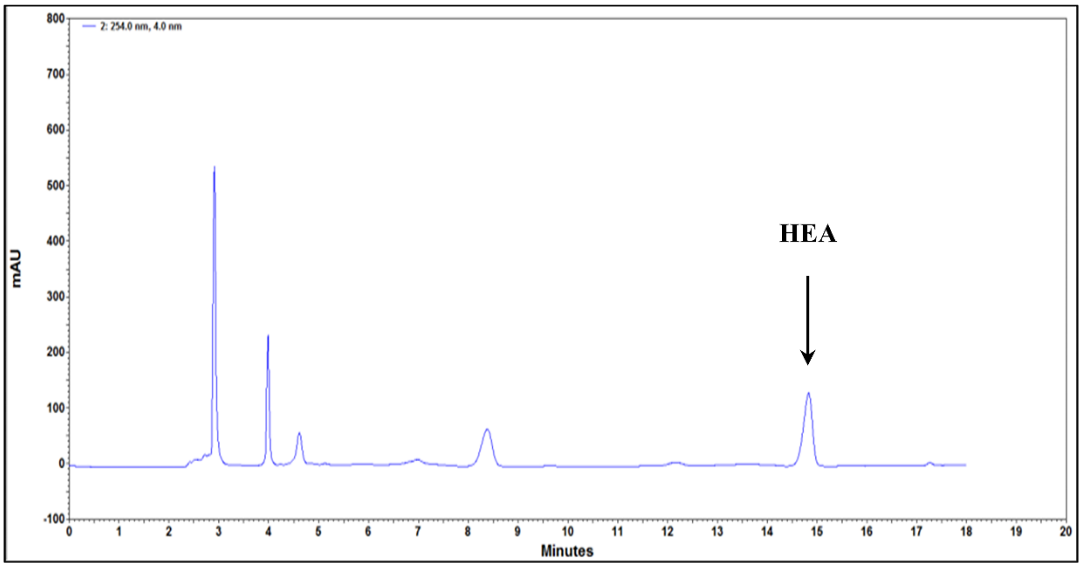

Supplementary Figure 1. HPLC of HEA extracted from CCM. Chemical analysis of the mycelium powder by high-performance liquid chromatography (HPLC). Two bioactive compounds, adenosine and N6-(2-hydroxyethyl) adenosine (HEA), were detected, with HEA showing a retention time of 14.9 minutes.
